# Entanglement and nonclassicality in four-mode Gaussian states generated via parametric down-conversion and frequency up-conversion

**DOI:** 10.1038/srep33802

**Published:** 2016-09-23

**Authors:** Ievgen I. Arkhipov, Jan Peřina Jr., Ondřej Haderka, Alessia Allevi, Maria Bondani

**Affiliations:** 1 RCPTM, Joint Laboratory of Optics of Palacký University and Institute of Physics of the Czech Academy of Sciences, 17. listopadu 12, 77146 Olomouc, Czech Republic; 2 Institute of Physics of CAS, Joint Laboratory of Optics of Palacký University and Institute of Physics, 17. listopadu 50a, 771 46 Olomouc, Czech Republic; 3 Dipartimento di Scienza e Alta Tecnologia, Università degli Studi dell’Insubria, Via Valleggio 11, 22100 Como, Italy; 4 Istituto di Fotonica e Nanotecnologie, Consiglio Nazionale delle Ricerche, Via Valleggio 11, 22100 Como, Italy

## Abstract

Multipartite entanglement and nonclassicality of four-mode Gaussian states generated in two simultaneous nonlinear processes involving parametric down-conversion and frequency up-conversion are analyzed assuming the vacuum as the initial state. Suitable conditions for the generation of highly entangled states are found. Transfer of the entanglement from the down-converted modes into the up-converted ones is also suggested. The analysis of the whole set of states reveals that sub-shot-noise intensity correlations between the equally-populated down-converted modes, as well as the equally-populated up-converted modes, uniquely identify entangled states. They represent a powerful entanglement identifier also in other cases with arbitrarily populated modes.

Since the discovery of quantum mechanics, entanglement has been considered a very peculiar and purely quantum feature of the physical systems. Its fundamental importance emerged when the experiments showing the violation of the Bell inequalities^1^,^2^,^3^, implementing quantum teleportation^4^,^5^ or demonstrating dense coding were performed. Nowadays, entanglement is undoubtedly considered as the key resource of modern and emerging quantum technology, including quantum metrology, quantum computation^6^ and quantum communications^7^,^8^,^9^.

For this reason, a great deal of attention has been devoted to the construction of practical sources of entangled light, both in the domains of discrete and continuous variables. While individual entangled photon pairs arising in spontaneous parametric down-conversion are commonly used in the discrete domain^10^, single-mode as well as two-mode squeezed states originating in parametric down-conversion and containing many photon pairs represent the sources in the domain of continuous variables^11^. Even more complex nonlinear optical processes, including those combining simultaneous parametric down-conversion and frequency up-conversion, have been analyzed as sources of more complex entangled states. This approach has been experimentally implemented in refs 12 and 13 considering three-mode entanglement and in ref. 14 where the four-mode entanglement has been analyzed.

Here, we consider a four-mode system composed of two down-converted modes and two up-converted modes. In the system, parametric down-conversion and frequency up-conversion involving both down-converted modes simultaneously occur in the same nonlinear medium^15^. While parametric down-conversion serves as the primary source of entanglement^16^, frequency up-conversion is responsible for the transfer of the entanglement to the up-converted modes.

This transfer operation is interesting from the fundamental point of view, as it generalizes the well-known property of ‘one-mode’ frequency up-conversion pumped by a strong coherent field, in which the statistical properties of the incident field are transferred to the frequency up-converted counterpart, also including the nonclassical ones (for example, squeezing,^17^). We note that such properties are important for the applications of the up-conversion process: For instance, it has been used many times for ‘shifting’ an optical ‘one-mode’ field to an appropriate frequency where its detection could be easily achieved^18^,^19^.

In the general analysis of the four-mode system, we quantify its global nonclassicality via the Lee nonclassicality depth^20^. Since the four-mode system under consideration cannot exhibit nonclassicality of individual single modes, the global nonclassicality automatically implies the presence of entanglement among the modes for a two-mode Gaussian system involving parametric down-conversion, (see ref. 21). The analysis of ‘the structure of entanglement’ further simplifies by applying the Van Loock and Furusawa inseparability criterion^22^ that excludes the presence of genuine three- and four-partite entangled states. This means that in the system discussed here there are only bipartite entangled states. It is thus sufficient to divide the analyzed four-mode state into different bipartitions to monitor the structure of entanglement. Then, the well-known entanglement criterion based on the positive partial transposition of the statistical operator^23^,^24^, which gives the logarithmic negativity as an entanglement quantifier, is straightforwardly applied^25^,^26^.

The experimental detection of two-mode (-partite) entanglement is in general quite challenging, as it requires measurements in complementary bases. Here, we theoretically show that, for the considered system with the assumed initial vacuum state, any two-mode partition exhibiting sub-shot-noise intensity correlations is also entangled. As a consequence, the measurement of intensity auto- and cross-correlations in this system is sufficient to give the evidence of the presence of two-mode entangled states through the commonly used noise reduction factor.

Finally, we note that the Hamiltonian of the analyzed four-mode system formally resembles that describing a twin beam with signal and idler fields divided at two beam splitters. This analogy results in similar properties of the four-mode states obtained in the two cases, though the processes of down-conversion and up-conversion occur simultaneously in our system, at variance with the system with two beam splitters, which modify the already emitted twin beam. We note that the system with two beam splitters has been frequently addressed in the literature as a prototype of more complex devices based on two multiports that are used to have access to intensity correlation functions for the detailed characterization of the measured fields^27^, also including their photon-number statistics^28^,^29^,^30^,^31^,^32^,^33^.

The paper is organized as follows. In Section *Four-mode nonlinear interaction* the model of four-mode nonlinear interaction including parametric down-conversion and frequency up-conversion is analyzed. Nonclassicality of the overall system is addressed in Section *Nonclassicality*. In Section *Four-mode entanglement*, the entanglement of the overall system is investigated considering the partitioning of the state into different bipartitions. Two-mode entangled states obtained after state reduction are analyzed in Section *Two-mode entanglement and noise reduction factor*, together with two-mode sub-shot-noise intensity correlations. Suitable parameters of the corresponding experimental setup can be found in Section *Experimental implementation*. Section *Conclusions* summarizes the obtained results.

## Four-mode nonlinear interaction

**Figure 1:**
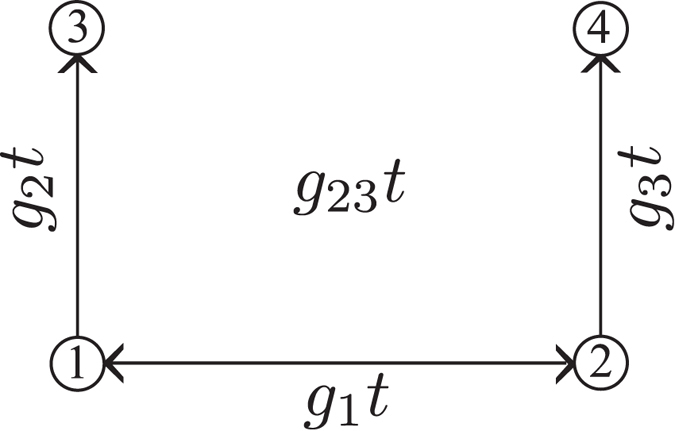
Optical fields in modes 1 and 2 interact via parametric down-conversion described by the nonlinear coupling constant g_1_. Photons from mode 1 (2) are converted into photons of mode 3 (4) thanks to the frequency up-conversion characterized by the coupling constant *g*_2_ (*g*_3_); *t* stands for the interaction time. In the symmetric case we have *g*_23_ = *g*_2_ = *g*_3_.

We consider a system of four nonlinearly interacting optical modes (for the scheme, see Fig. 1). Photons in modes 1 and 2 are generated by parametric down-conversion with strong pumping (coupling constant *g*_1_). Photons in mode 1 (2) can then be annihilated with the simultaneous creation of photons in mode 3 (4). The two up-conversion processes are possible thanks to the presence of two additional strong pump fields with coupling constants *g*_2_ and *g*_3_. The overall interaction Hamiltonian for the considered four-mode system is written as^15^:


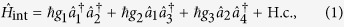


where the operators 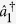 and 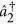 create an entangled photon pair in modes 1 and 2 and the creation operators 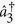 and 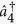 put the up-converted photons into modes 3 and 4, respectively. Symbol H.c. replaces the Hermitian conjugated terms.

The Heisenberg-Langevin equations corresponding to the Hamiltonian 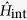 in Eq. (1) are written in their matrix form as follows:


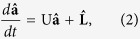


where 
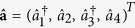
 and 
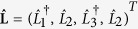
. The matrix **U** introduced in Eq. (2) is expressed as


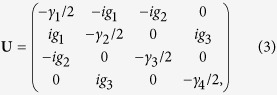


in which *γ*_*j*_ stands for the damping coefficient of mode *j*, *j* = 1, …, 4. The Langevin operators 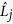, *j* = 1, …, 4, obey the following relations:


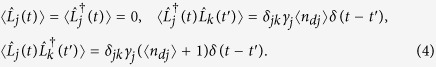


The Kronecker symbol is denoted as *δ*_*ij*_ and the symbol *δ*(*t*) means the Dirac function. The mean numbers *n*_*dj*_ corresponding to noise reservoir photons have been used in Eq. (4). We note that for the noiseless system the following quantity 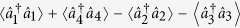 is conserved in the interaction.

Introducing frequencies *ω*_*j*_ and wave vectors 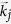 of the mutually interacting modes, we formulate the assumed ideal frequency and phase-matching conditions of the considered nonlinear interactions in the form:


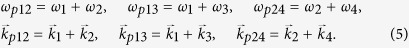


In Eq. (5), *ω*_*p*12_ (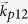) stands for the pump-field frequency (wave vector) of parametric down-conversion, whereas *ω*_*p*13_ [*ω*_*p*24_] 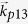 [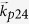]) means the frequency (wave vector) of the field pumping the up-conversion process between modes 1 [2] and 3 [4].

The solution of the system of first-order linear operator stochastic equations (2) can be conveniently expressed in the following matrix form:


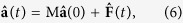


where the evolution matrix **M** is written in Eq. (18) of Appendix for the noiseless case and vector 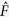 arises from the presence of the stochastic Langevin forces. More details can be found in ref. 34. When applying the solution (6), we consider the appropriate phases of the three pump fields such that the coupling constants *g*_*j*_, *j* = 1, 2, 3, are real.

The statistical properties of the optical fields generated both by parametric down-conversion and up-conversion are described by the normal characteristic function 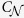 defined as


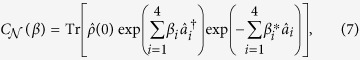


where Tr denotes the trace and *β* ≡ (*β*_1_, *β*_2_, *β*_3_, *β*_4_)^*T*^. Using the solution given in Eq. (6), the normal characteristic function 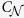 attains the Gaussian form:


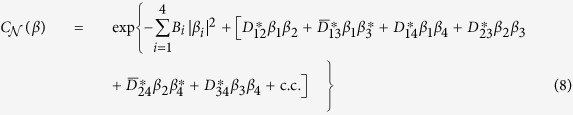


and c.c. replaces the complex conjugated terms. The coefficients occurring in Eq. (8) are derived in the form:


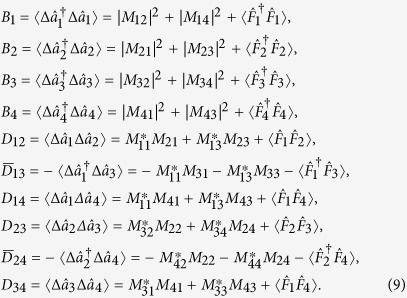


We note that the two-mode interactions characterized by the coefficients *D*_*ij*_ and 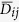 in Eq. (8) attain specific forms. While the coefficients *D*_*ij*_ reflect the presence of photon pairs in modes *i* and *j*, coefficients 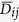 describe mutual transfer of individual photons between modes *i* and *j*.

The normal characteristic function 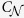 can be rewritten in the matrix form exp(*β*^†^**A***β*/2) by introducing the normally-ordered covariance matrix **A**:


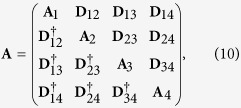


where the 2 × 2 matrices are defined as:


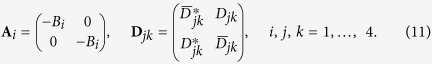


The covariance matrix *σ* related to the symmetric ordering and corresponding to the phase space 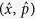 is needed to perform easily partial transposition. It has the same structure as the covariance matrix **A** written in Eq. (10) with the blocks **A**_*i*_ (**D**_*jk*_) replaced by the blocks *σ*_*i*_ (*ε*_*jk*_) defined as:


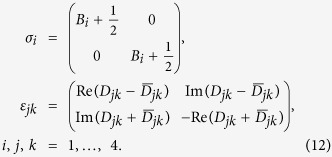


Symbol Re (Im) denotes the real (imaginary) part of the argument.

In what follows, we consider the situation in which all four modes begin their interaction in the vacuum state. Moreover, we focus on the specific symmetric case in which *g*_2_ = *g*_3_ ≡ *g*_23_. A note concerning the general case *g*_2_ ≠ *g*_3_ is found at the end.

## Nonclassicality

We first analyze the global nonclassicality of the whole four-mode system as it is relatively easy and, for the considered initial vacuum state, it implies entanglement (see below). Nonclassicality of the whole four-mode state described by the statistical operator 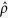 is conveniently quantified by the Lee nonclassicality depth *τ*^20^. This quantity gives the amount of noise, expressed in photon numbers, needed to conceal nonclassical properties exhibited by the Glauber-Sudarshan *P* function, which attains negative values in certain regions or even does not exist as an ordinary function. The Glauber-Sudarshan *P* function is determined by the Fourier transform of the normally-ordered characteristic function 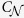 given in Eq. (8). Technically, the Lee nonclassicality depth is given by the largest positive eigenvalue of the covariance matrix **A** defined in Eq. (10). So, it can be easily determined.

**Figure 2:**
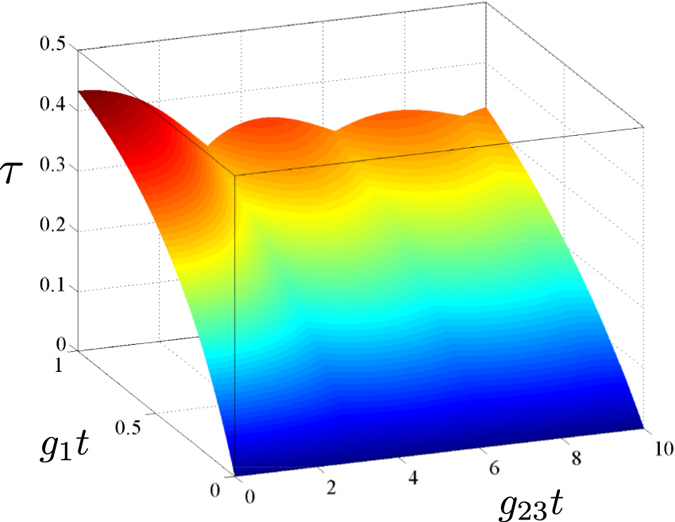
Nonclassicality depth *τ* as a function of the parameters *g*_1_*t* and *g*_23_*t*.

The Lee nonclassicality depth *τ* as a function of the coupling parameters *g*_1_*t* and *g*_23_*t* is shown in Fig. 2. The increasing values of *g*_1_*t* result in larger values of the nonclassicality depth *τ*, as the number of photons simultaneously generated in modes 1 and 2 increases. We note that this pairing of photons in the process of parametric down-conversion is the only source of nonclassicality in the analyzed four-mode system. On the contrary, nonzero values of parameter *g*_23_*t* only lead to the oscillations of the nonclassicality depth *τ*. This behavior occurs as the frequency up-conversion moves photons, and so also photon pairs, from modes 1 and 2 to modes 3 and 4 and vice versa (see the scheme in Fig. 1). This results in the nonclassical properties of modes 3 and 4, at the expenses of the nonclassical properties of modes 1 and 2.

The maximum value of the Lee nonclassicality depth *τ* = 0.5 is reached for *g*_23_*t* = 0 and ideally in the limit *g*_1_*t* → ∞, i.e. when only the strong parametric down-conversion occurs. This is in agreement with the analysis of nonclassical properties of twin beams reported in ref. 35. The value *τ* = 0.5 can also be asymptotically reached in the limit *g*_23_*t* → ∞, in which we have


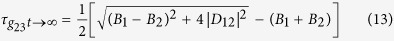


with *B*_3_ → *B*_1_, *B*_4_ → *B*_2_ and *D*_34_ → *D*_12_. It is worth noting that formula (13) applies also for *g*_23_*t* = 0.

**Figure 3:**
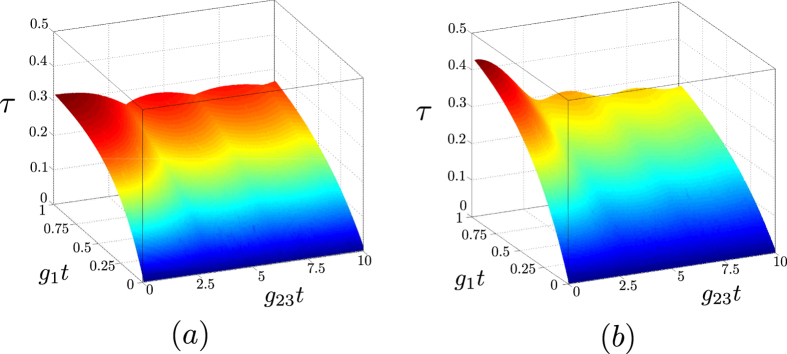
Nonclassicality depth *τ* as a function of the parameters *g*_1_*t* and *g*_23_*t* for (**a**) *γ*_1_*t* = *γ*_2_*t* = *g*_1_*t*, *γ*_3_*t* = *γ*_4_*t* = 0; (**b**) *γ*_1_*t* = *γ*_2_*t* = 0, *γ*_3_*t* = *γ*_4_*t* = *g*_1_*t*, assuming *n*_*dj*_ = 0 for *j* = 1, …, 4.

Nonclassicality is also strongly resistant against damping in the system. This means that even a low number of photon pairs is sufficient to have a nonclassical state. We demonstrate this resistance by considering the damping constants *γ* proportional to the nonlinear coupling constant *g*_1_, which quantifies the speed of photon-pair generation. The graphs in Fig. 3 show that the generated states remain strongly nonclassical even though a considerable fraction of photon pairs is broken under these conditions. The comparison of graphs in Fig. 3(a,b) reveals that the damping is more detrimental in the down-converted modes 1 and 2 than in the up-converted modes 3 and 4.

At variance with nonclassicality, the determination and quantification of entanglement is more complex and it is technically accomplished by considering all possible bipartitions of the four-mode system (see the next Section). On the one side all bipartitions considered below are in principle sufficient to indicate entanglement, on the other side the application of the Van Loock and Furusawa inseparability criterion^22^ to our system excludes the presence of genuine three- and four-mode entanglement. The analyzed Hamiltonian written in Eq. (1) together with the incident vacuum state also excludes the presence of nonclassical states in individual modes. In what follows, the bipartite entanglement is thus the only source of the global nonclassicality in the analyzed system. This situation considerably simplifies the possible experimental investigations as positive values of the Lee nonclassicality depth directly imply the presence of entanglement somewhere in the system.

## Four-mode entanglement

In quantifying the entanglement in our four-mode Gaussian system, we rely on the following facts applicable to an arbitrary (*m* + *n*)-mode Gaussian state. It has been proven that positivity of the partially transposed (PPT) statistical operator describing any 2 × 2 or 2 × 3 bipartition of the state is a necessary condition for the separability of the state^23^,^24^. Moreover, it has been shown that the violation of PPT condition occurring in any 1 × (*m* + *n* − 1) bipartitions or *m* × *n* bisymmetric bipartitions for *m* > 2 and *n* > 3 is a sufficient condition for the entanglement in the analyzed (*m* + *n*)-mode state^36^,^37^. For continuous variables systems, the PPT is simply accomplished when the symmetrically-ordered field operators are considered allowing to perform the PPT only by changing the signs of the momenta 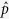
36. Moreover, symplectic eiganvalues 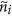 of the symmetrically-ordered covariance matrix *σ* can be conveniently used to quantify entanglement in bipartite systems via the logarithmic negativity *E*^26^, defined in terms of eigenvalues 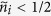:


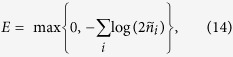


where max gives the maximal value.

In the four-mode Gaussian state sketched in Fig. 1, we have two kinds of bipartitions. Either a single mode forms one subsystem and the remaining three modes belong to the other subsystem, or two modes are in one subsystem and the remaining two modes lie in the other subsystem. Due to the symmetry, only two members of each group are of interest for us. Namely, these are bipartitions 1 × 234 and 3 × 124 from the first group and bipartitions 12 × 34 and 13 × 24 from the second one. We note that, while the bipartition 12 × 34 is bisymmetric in our interaction configuration (provided that *g*_2_*t* = *g*_3_*t*), the bipartition 13 × 24 is not bisymmetric. Nevertheless, positive values of both the logarithmic negativities *E*_12×34_ and *E*_13×24_ reflect entanglement as both bipartitions involve two modes on both sides. Similarly, positive values of the logarithmic negativities *E*_1×234_ and *E*_3×124_ guarantee the presence of entanglement.

**Figure 4:**
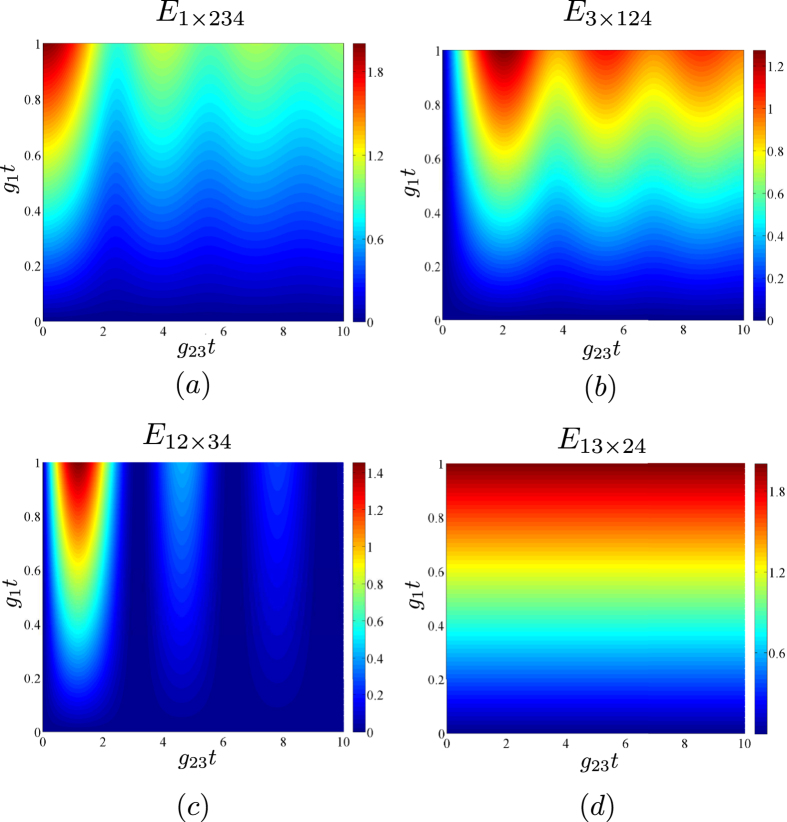
Logarithmic negativities *E*_1×234_ (**a**), *E*_3×124_ (**b**), *E*_12×34_ (**c**), and *E*_13×24_ (**d**) as functions of parameters *g*_1_*t* and *g*_23_*t* for different bipartitions indicated in the subscripts.

We first pay attention to the entanglement expressed in the logarithmic negativities *E*_1×234_ and *E*_3×124_. As suggested by the graphs in Fig. 4(a,b), the oscillating behavior of negativity *E*_1×234_ is complementary to that of negativity *E*_3×124_. This means that the larger values of negativity *E*_1×234_ are accompanied by the lower values of negativity *E*_3×124_ and vice versa. Such a result is a consequence of the fact that the entanglement is due to the presence of photon pairs and a photon created in mode 1 can move to mode 3 and later return back to mode 1. This movement leads to the oscillations with frequency *g*_23_, which are clearly visible in Fig. 4(a,b). This explanation also suggests that no entanglement is possible between modes 1 and 3. Indeed, if we also determine the negativity *E*_1×24_ (or *E*_3×24_), we will get the same values already obtained for the negativity *E*_1×234_ (*E*_3×124_).

The negativity *E*_12×34_, characterizing the entanglement between the twin beam in modes 1 and 2 and the up-converted beams in modes 3 and 4, is plotted in Fig. 4(c). It reflects the gradual movement of photon pairs from modes 1 and 2, where they are created, to modes 3 and 4. Note that the maxima of negativity *E*_12×34_ along the *g*_23_*t*-axis occur inbetween the maxima of negativities *E*_1×234_ and *E*_3×124_. The origin of entanglement in photon pairing is confirmed in the graph of Fig. 4(d), showing that the negativity *E*_13×24_ is independent of parameter *g*_23_*t* and that the negativity *E*_13×24_ increases with the increasing parameter *g*_1_*t*. In certain sense, the independence of negativity *E*_13×24_ from parameter *g*_23_*t* represents the conservation law for nonclassical resources, as the negativities of the different two-mode reductions derived from this bipartition (*E*_1×2_, *E*_1×4_, *E*_3×2_, and *E*_3×4_) do depend on parameter *g*_23_*t*.

**Figure 5:**
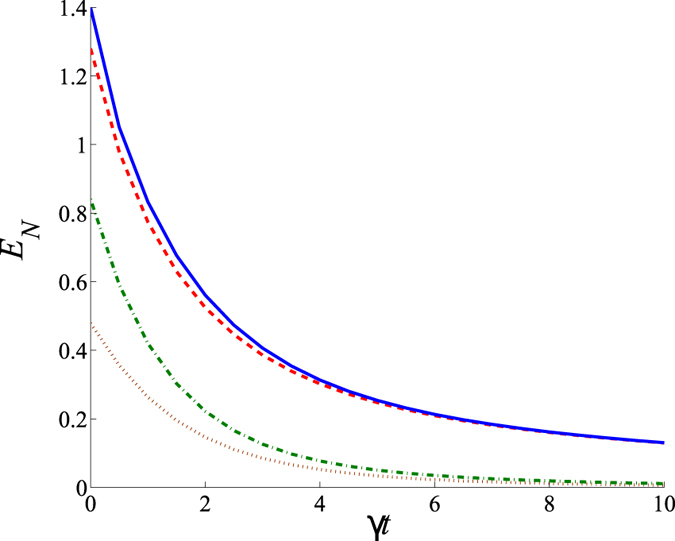
Logarithmic negativity *E* as a function of the damping coefficient *γt* for different bipartitions: 1 × 234 (dashed red line), 3 × 124 (brown dotted line), 12 × 34 (dashed-dotted green line), and 13 × 24 (solid blue line). We set *g*_1_*t* = *g*_2_*t* = *g*_3_*t* = 0.7, *γ* ≡ *γ*_1_ = *γ*_2_ = *γ*_3_ = *γ*_4_; *n*_*dj*_ = 0 for *j* = 1, …, 4.

The developed model also allows us to study the role of damping in the entanglement creation. The investigations based on equal damping constants *γ* and noiseless reservoirs (*n*_*d*_ = 0) just reveal the deterioration of entanglement in all the considered bipartitions with the increase of damping constants see (Fig. 5).

## Two-mode entanglement and noise reduction factor

The results of the theoretical analysis suggest that, from the experimental point of view, the observation of entanglement between pairs of modes is substantial for the characterization of the emitted entangled states. Formally, the theory describes such observations through the reduced two-mode statistical operators. The analysis shows that the behavior of two-mode negativities *E*_1×2_, *E*_3×4_, and *E*_1×4_ with respect to parameters *g*_1_*t* and *g*_23_*t* is qualitatively similar to that of four-mode negativities *E*_1×234_, *E*_3×124_, and *E*_12×34_ plotted in Fig. 4(a–c). This similarity originates in possible ‘trajectories’ of photon pairs born in modes 1 and 2 and responsible for the entanglement.

**Figure 6:**
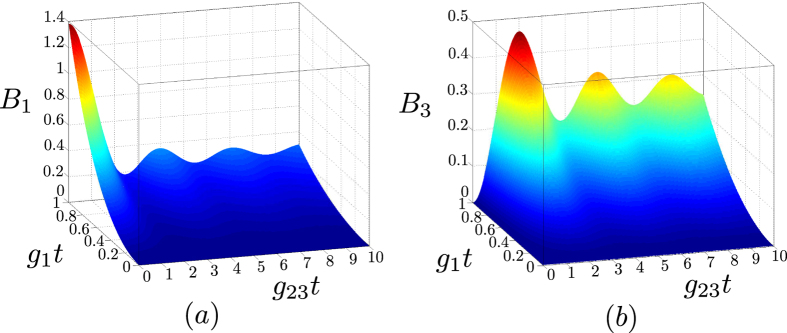
Mean photon numbers *B*_1_ (**a**) and *B*_3_ (**b**) plotted as functions of parameters *g*_1_*t* and *g*_23_*t*.

Additional insight into the generation of entanglement in the analyzed system is provided when the entanglement is related to the intensities of the interacting fields. As quantified in the graphs of Fig. 6, both mean photon numbers *B*_1_ ≡ *B*_2_ and *B*_3_ ≡ *B*_4_ are increasing functions of parameter *g*_1_*t* and oscillating functions of parameter *g*_23_*t*. This oscillating behavior is particularly interesting, as it reflects the flow of photons from modes 1 and 2 to modes 3 and 4, respectively, and vice versa. As we will see below, this is in agreement with the ‘flow of the entanglement’ among the modes.

**Figure 7:**
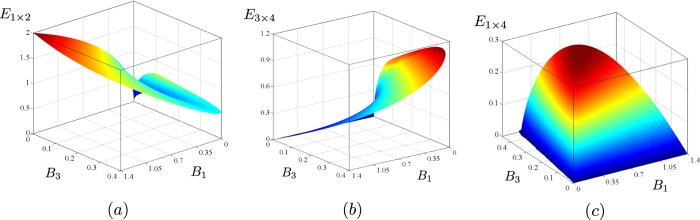
Logarithmic negativities *E*_1×2_ (**a**), *E*_3×4_ (**b**) and *E*_1×4_ (**c**) as functions of the mean photon numbers *B*_1_ and *B*_3_.

The graph in Fig. 7(a) shows that the negativity *E*_1×2_ is on the one side an increasing function of the mean photon number *B*_1_, on the other side it only weakly depends on the mean photon number *B*_3_. This confirms that pairing of photons in parametric down-conversion is the only resource for entanglement creation. On the contrary, as shown in Fig. 7(b), the negativity *E*_3×4_ is an increasing function of the mean photon number *B*_3_, whereas it weakly depends on the mean photon number *B*_1_. This indicates that the entanglement in modes 34 comes from modes 12 through the transfer of photon pairs: The stronger the transfer is, the larger the value of negativity *E*_3×4_ is. Moreover, optimal conditions for the observation of entanglement in modes 1 and 4 occur provided that there is the largest available number of photon pairs with one photon in mode 1 and its twin in mode 4. According to the graph in Fig. 7(c) this occurs when the mean photon numbers *B*_4_ (*B*_4_ ≡ *B*_3_) and *B*_1_ are balanced, independently of their values.

In general, the experimental identification of two-mode entanglement is not easy, as it requires the simultaneous measurement of the entangled state in two complementary bases. Alternatively, entanglement can be inferred from the reconstructed two-mode phase-space quasi-distribution, which needs two simultaneous homodyne detectors^38^, each one endowed with a local oscillator. However, the detection of entanglement, at least in some cases, can be experimentally accomplished by the observation of sub-shot-noise intensity correlations. This is a consequence of the detailed numerical analysis, which reveals that the majority of the reduced two-mode entangled states also exhibits sub-shot-noise intensity correlations. Nevertheless, it should be emphasized here that, in the analyzed system, there are also two-mode entangled states not exhibiting sub-shot-noise intensity correlations. On the contrary, we note that the reduced two-mode separable states do not naturally exhibit sub-shot-noise intensity correlations.

Sub-shot-noise intensity correlations are quantified by the noise reduction factor *R*^39^,^40^, that is routinely measured to recognize nonclassical intensity correlations of two optical fields. The noise reduction factor *R* expressed in the moments of photon numbers *n*_*j*_ and *n*_*k*_ of modes *j* and *k*, respectively, is defined by the formula:


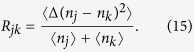


Sub-shot-noise intensity correlations are described by the condition *R* < 1. We note that there exists the whole hierarchy of inequalities involving higher-order moments of photon numbers (or intensities)^27^,^33^,^41^,^42^ that indicate nonclassicality and, in our system, also entanglement. We mention here the inequality derived by Lee^43^ as a practical example that is sometimes used in the experimental identification of nonclassicality. We note that this criterion is stronger than the noise reduction factor *R* in revealing the nonclassicality^39^.

**Figure 8:**
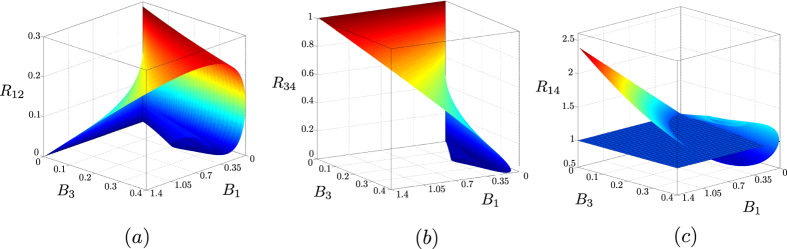
Noise reduction factors *R*_1×2_ (**a**), *R*_3×4_ (**b**) and *R*_1×4_ (**c**) as functions of the mean photon numbers *B*_1_ and *B*_3_. In (**c**), the plane defined as *R*_1×4_ = 1 is represented by the blue mesh.

**Figure 9:**
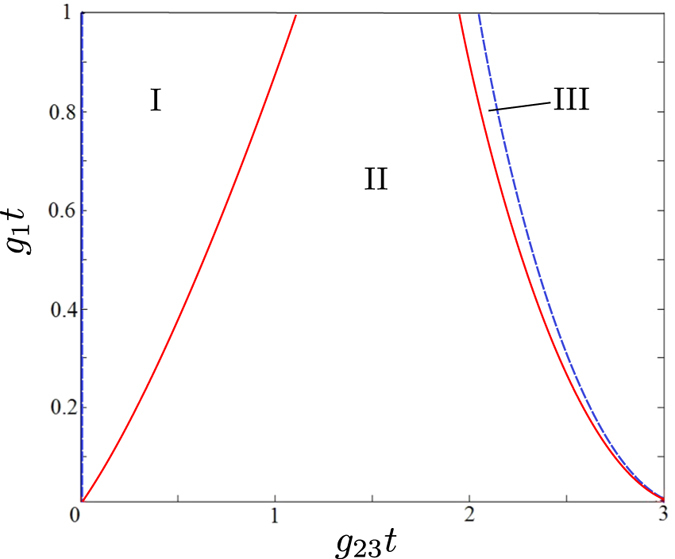
Solutions of the equations for logarithmic negativity *E*_1×4_ = 0 (blue dashed line) and noise reduction factor *R*_1×4_ = 1 (red solid line) in the plane spanned by parameters g_1_*t* and g_23_*t*. The two-mode field is entangled (*E*_1×4_ > 0) inbetween the blue dashed lines, i.e. in the areas I, II, and III, whereas it is sub-shot-noise (*R*_1×4_ < 1) in between the red solid lines, i.e. in the area II.

The noise reduction factors *R*_1×2_, *R*_3×4_ and *R*_1×4_ describing the reduced two-mode fields with their negativities plotted in Fig. 7 are drawn in Fig. 8 for comparison. We can see complementary behavior of the negativities *E* and noise reduction factors *R* in the graphs in Figs 7 and 8. An increase of the negativity *E* is accompanied by a decrease in the noise reduction factor *R*. A closer inspection of the curves in these graphs shows that the condition *R* < 1 identifies very well entangled states when the noise reduction factor is measured in modes 1 × 2 and 3 × 4. Nevertheless, there are entangled states with *R*_1×4_ > 1, as shown in the graph of Fig. 9, in which the values of parameters *g*_1_*t* and *g*_23_*t* appropriate for this situation occur in the areas I and III. On the other hand, the entangled states found in the area II in the graph of Fig. 9 have *R* < 1. It is worth noting that the relative amount of entangled states not detected via *R* < 1 increases with the increasing coupling constant *g*_1_*t* and so with the increasing overall number of photons in the system.

The observed relation between the entangled states and those exhibiting sub-shot-noise intensity correlations can even be explained theoretically, due to the specific form of the reduced two-mode Gaussian states analyzed in ref. 35. According to ref. 35 entangled states in modes *i* and *j* are identified through the inequality *B*_*i*_*B*_*j*_ < |*D*_*ij*_|^2^. On the other hand, the noise reduction factor *R*_*ij*_ defined in Eq. (15) attains for our modes the form:


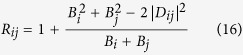


that assigns the sub-shot-noise intensity correlations to the states obeying the inequality 
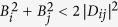
. Thus, the inequality 
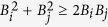
 implies that the states with sub-shot-noise intensity correlations form a subset in the set of all entangled states. Moreover, if *B*_*i*_ = *B*_*j*_, both sets coincide as we have 
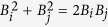
. Thus, the noise reduction factors *R*_12_ and *R*_34_ are reliable in identifying entangled states in the symmetric case, in which *B*_1_ = *B*_2_ and *B*_3_ = *B*_4_.

We note that, according to the theory developed for the modes without an additional internal structure^35^, the logarithmic negativity *E*_*ij*_ can be determined along the formula^35^


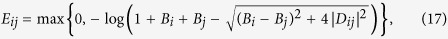


where |*D*_*ij*_|^2^ = 〈Δ*n*_*i*_Δ*n*_*j*_〉. According to Eq. 17 the logarithmic negativity *E*_*ij*_ can, in principle, be inferred from the measured mean intensities in modes *i* and *j* and the cross-correlation function of intensity fluctuations in this idealized case.

At the end, we make a note about the entanglement in the general four-mode system with different up-conversion coupling constants (*g*_2_ ≠ *g*_3_). This is relevant when non-ideal phase-matching conditions of the three nonlinear interactions are met in the experiment (see below). According to our investigations, the largest values of negativities *E*_1×2_ and *E*_3×4_ are found in the symmetric four-mode system (*g*_2_ = *g*_3_) considered above. On the contrary, the largest values of negativities *E*_1×4_ and *E*_2×3_ are obtained for unbalanced *g*_2_ and *g*_3_ interactions.

**Figure 10:**
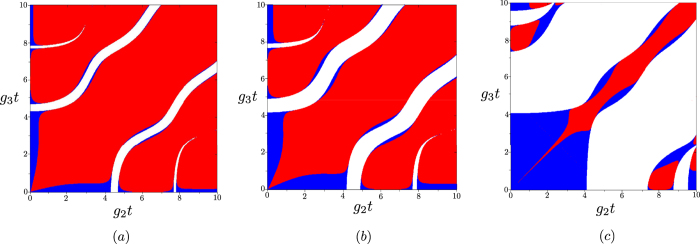
Planes given by *g*_1_*t* = 0.5 (**a**), *g*_1_*t* = 1 (**b**) and *g*_1_*t* = 5 (**c**) in the ‘phase diagram’ identifying classical states (white areas), entangled states without sub-shot-noise intensity correlations (blue) and entangled states with sub-shot-noise intensity correlations (red) in the space spanned by the coupling constants *g*_*j*_*t*, *j* = 1, 2, 3.

Similarly to the symmetric case, separable states, entangled states without sub-shot-noise intensity correlations and entangled states exhibiting sub-shot-noise intensity correlations are found in the whole three-dimensional parametric space spanned by variables *g*_*j*_*t* for *j* = 1, 2, 3. As an example, the distribution of different kinds of reduced two-mode states found in the up-converted modes 3 and 4 in this space is plotted in Fig. 10. The graphs in Fig. 10 indicate that, in accord with the symmetric case, the larger the value of constant *g*_1_*t*, the larger the relative amount of entangled states that cannot be identified through sub-shot-noise intensity correlations.

## Experimental implementation

A possible experimental implementation of the four mode interaction described above can be achieved by using a BaB_2_O_4_ crystal as the nonlinear medium, a ps-pulsed laser (a mode-locked Nd:YLF laser regeneratively amplified at 500 Hz, High-Q Laser Production) to get the pump fields and hybrid photodetectors (mod. R10467U-40, Hamamatsu Photonics) as the photon-number-resolving detectors. A typical experimental setup can be built in analogy with other previous experiments^33^. The phase-matching conditions can be chosen so as to have *ω*_1_ = *ω*_2_ and a common pump field for both up-conversion processes so that *ω*_3_ = *ω*_4_. In this specific symmetric case we have *g*_2_ = *g*_3_ ≡ *g*_23_.

We can estimate the range of coupling constants achievable in this setup based on the above-mentioned laser source. Let us consider the following parameters: wavelength of the pump for down-conversion *λ*_*p*1_ = 349 nm, *λ*_1_ = *λ*_2_ = 698 nm, wavelength of the pump for up-conversion *λ*_*p*2_ = 1047 nm, *λ*_3_ = *λ*_4_ = 418.8 nm, length of the BaB_2_O_4_ crystal *L* = 4 mm, diameters of the pumps 0.5 mm, pulse duration 4.5 ps. The coupling constants *g*_1_ and *g*_23_ are linearly proportional to the corresponding pump field amplitudes so that *g*_1_*t* = *κ*_1_*A*_*p*1_*L* and *g*_23_*t* = *κ*_23_*A*_*p*2_*L*, where *κ*_*j*_ (*j* = 1, 23) are the nonlinear coupling coefficients and *A*_*j*_ (*j* = *p*1, *p*2) are the pump amplitudes. For the considered parameters we can estimate *κ*_*j*_ ≈ 10^−13^*s*^1/2^. The useful range of energies per pulse is up to 66 *μ*J in the UV and up to 240 *μ*J in the IR, corresponding to maximum values *g*_1_*t* ≈ 5.9 and *g*_2_*t* ≈ 7. The theoretical results discussed above predict an interesting behavior for this range of parameters, including the transfer of entanglement into the up-converted modes.

## Conclusions

Four-mode Gaussian states generated via parametric down-conversion and frequency up-conversion have been analyzed in terms of nonclassicality, entanglement and entanglement transfer among the modes. While nonclassicality of the state has been described by the easily-computable Lee nonclassicality depth, logarithmic negativity for different bipartitions has been applied to monitor the occurrence of entanglement among different modes. It has been shown that whenever the analyzed system is nonclassical, it is also entangled. Moreover, the entanglement is present only in the form of bipartite entanglement. The analysis of the noise reduction factor identifying sub-shot-noise intensity correlations, in parallel with the logarithmic negativity quantifying two-mode entanglement, has shown that the noise reduction factor is a powerful indicator of the entanglement in the analyzed system. This is substantial for the experimental demonstration of the transfer of entanglement from the down-converted modes to the up-converted ones.

## The evolution matrix M

The evolution matrix **M** describing the operator solution of the Heisenberg equations written in Eq. (2) is derived in the form:


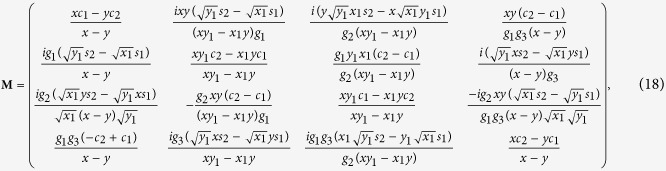


where *x* = (*a* + *b*)/2, *x*_1_ = (*a*_1_ + *b*)/2, *y* = (*a* − *b*)/2, *y*_1_ = (*a*_1_ − *b*)/2, 
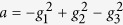
, 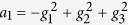, 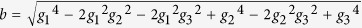, 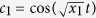, 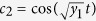, 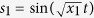, and 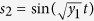.

## Additional Information

**How to cite this article**: Arkhipov, I. I. *et al*. Entanglement and nonclassicality in four-mode Gaussian states generated via parametric down-conversion and frequency up-conversion. *Sci. Rep.* **6**, 33802; doi: 10.1038/srep33802 (2016).
